# Association of Dietary Inflammation Index and *Helicobacter pylori* Immunoglobulin G Seropositivity in US Adults: A Population-Based Study

**DOI:** 10.1155/2023/8880428

**Published:** 2023-07-28

**Authors:** Lin Shi, Dan Zhang

**Affiliations:** ^1^Department of Gastroenterology, Xuzhou Central Hospital, Xuzhou Clinical School of Xuzhou Medical University, Xuzhou, Jiangsu, China; ^2^Department of Nephrology, Affiliated Hospital of Xuzhou Medical University, 99 West Huai-hai Road, Xuzhou 221002, Jiangsu, China

## Abstract

**Background:**

Dietary patterns play important role in *Helicobacter pylori* (*H. pylori*) infection. We aimed to investigate the potential relationship between Dietary Inflammation Index (DII) and *H. pylori* infection in US adults.

**Methods:**

This cross-sectional study was based on National Health and Nutrition Examination Survey (1999–2000). Individuals aged ≥20 years who provided a 24 hr dietary intake history and underwent *H. pylori* testing were included in the analysis. Multivariate weighted logistic regression analysis, smooth curve fitting, and subgroup analysis were used to investigate the relationship between DII and *H. pylori* infection. Subgroup analyses were based on demographic and clinical variables.

**Results:**

There were 4,000 individuals enrolled in our final analysis. The overall mean age was 45.92 years and 46.77% were males. The overall prevalence of *H. pylori* infection in the study population was 45.9%. The smooth curve fitting analysis indicated a near-linear relationship between DII and *H. pylori*. In multivariate weighted logistic regression analysis, the odds ratio (OR) of DII is 1.17 (95% confidence interval (CI), 1.09–1.27) for *H. pylori* infection. In subgroup analysis, DII still increased the risk of *H. pylori* infection independently.

**Conclusions:**

The increased DII levels were associated with an increased risk of *H. pylori* infection among US adults. Further studies are needed to elucidate the exact mechanisms of DII and *H. pylori* infection.

## 1. Introduction

*Helicobacter pylori* (*H. pylori*) is a Gram-negative, transmissible bacterium that colonizes gastric mucosa and infects the stomachs of nearly 4.4 billion people around the world [1]. In Hong Kong and neighbouring regions including Mainland China, Japan, South Korea and Taiwan, the infection rate of *Helicobacter pylori* in 2017 exceeded 50% [2]. Some scholars have proposed that *H. pylori* can be transmitted through oral or fecal oral routes, resulting in family transmission [3]. The infection is usually acquired in childhood, can lead to long-term or even lifelong chronic gastritis, about 10% of infected people eventually develop clinical manifestations, such as atrophic gastritis, erosive gastritis, peptic ulcer, and even gastric cancer or mucosa-associated lymphoid tissue (MALT) lymphoma [1]. *H. pylori* can also directly or indirectly affect intestinal flora and brain-gut axis by promoting the release of inflammatory mediators and cytokines [4].

In addition to some of the factors that have been found to be susceptible to *H. pylori*, such as age, poor living environment and bad habits, and nutrition and dietary factors have also become more and more important [3, 5]. It has been reported that a balanced diet, especially a diet rich in fruits and vegetables, helps to reduce the incidence of *H. pylori* infection. in addition, a diet rich in carbohydrate or too sweet may increase that rate of *H. pylori* infection [5]. Moreover, in a case–control study conducted in Iran, researchers found that a dietary pattern rich in antioxidants such as vitamin *A*, *C*, and *E* may help reduce the risk of *H. pylori* infection [3].

In recent years, the relationship between diet and chronic diseases has been paid more and more attention, especially the inflammatory diet pattern has been proved to be related to many chronic diseases such as chronic kidney disease, depression and rheumatoid arthritis [6–8]. The Dietary Inflammatory Index (DII) is a measure of the inflammatory potential of a diet. It is based on the nutrient content of foods and is designed to provide an overall measure of dietary inflammatory potential [9]. The DII is calculated by assigning a score to each food based on its content of nutrients associated with either proinflammatory or anti-inflammatory effects. The scores of all the foods in a diet are then combined to give an overall score for the diet. Therefore, DII could reflect the inflammation in a person's diet as a whole and give a comprehensive score for whether a diet is anti- or proinflammatory [10, 11]. To our knowledge, there are few studies on DII and *H. pylori* infection. In our study, we aimed to examine the association between DII and the risk of HP infection after adjustment for potential confounders, and to investigate the stability of the association in different subgroups of the population.

## 2. Materials and Methods

### 2.1. Data Acquisition and Study Individuals

The entire data of our study was collected from the National Health and Nutrition Examination Survey (NHANES) for the years 1999–2000. The NHANES is a continuous survey created and conducted by the National Center for Health Statistics (NCHS) of the Centers for Disease Control and Prevention (CDC) [12]. A complicated, multistage, stratified, clustered probability technique was utilized to choose a representative sample of Americans [13–15]. The CDC's Institutional Review Board has approved the protocol for the survey. Each participant has giver their informed consent form in NHANES. During the NHANES 1999–2000, people aged 20 or older who participated in consecutive study cycles were examined. In our analysis, the exclusion criteria were participants without sufficient data to calculate DII or without *H* pylori immunoglobulin G information [16–19].

**Figure 1:**
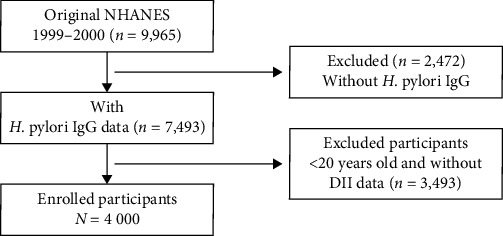
Flow chart of sample selection from the NHANES 1999–2000.

A total of 9,965 participants was enrolled in the first step in 1999–2000 cycle; after the exclusion of participants with missing data about *H. pylori* antibody (*n* = 2,472), DII information and aged less than 20 years old (3,493), 4,000 eligible participants aged ≥20 years were included in our final analysis (Figure 1).

### 2.2. Dietary Intake Assessment and DII

Diet-related nutritional and energy intake data were obtained through two 24 hr dietary recall interviews, conducted by experienced and trained dietitians with a previously validated food frequency questionnaire (FFQ) [8]. The Food and Nutrient Database for Dietary Studies (FNDDS) was used to estimate diet nutrient and energy intake. Interviewers employed the Automated Multichannel Method of the United States Department of Agriculture to collect more accurate and comprehensive data for the NHANES study [20, 21].

The details of calculating DII have been described elsewhere. The impact of diet on inflammation was evaluated using 45 dietary components, such as macronutrients, micronutrients, other active elements, and ten whole foods. A database with 11 countries for each of the 45 parameters was used to normalize each individual's intakes of each food parameter to global intakes based on each parameter's mean and standard deviation [22]. The standardized intake scores (*Z* scores) were transformed into proportions and centered. The centered proportions of these specific food intakes were multiplied by their inflammatory effect scores and summed to get an overall DII score for the person's diet. In the current study, 28 food parameters were employed to calculate DII, which were as follows: carbohydrates, protein, total fat, alcohol, fiber, cholesterol, saturated fatty acids, monounsaturated fatty acids, polyunsaturated fatty acids, *n*−3 fatty acids, *n*−6 fatty acids, niacin, iron, magnesium, zinc, selenium, folic acid, carotene, caffeine, vitamin A, thiamine, vitamin B2, vitamin B6, vitamin B12, vitamin C, vitamin D, vitamin E, and energy [6–8]. It should be noted that although the original DII calculation required 45 dietary components, only 28 were available in the NHANES database (Table S1), but the DII index based on 28 dietary components is well documented and reflects dietary inflammation well, and 28 food components-based DII has been widely used in research [6, 8, 22]. Detailed methods and process of DII calculation were provided in the supplementary material.

### 2.3. Assessment of H Pylori Infection

The *H. pylori* IgG enzyme-linked immunosorbent assays (ELISA) from Wampole Laboratories (Wampole) are designed to detect and quantitatively determine the concentration of IgG antibodies to *Helicobacter pylori* in human serum [23]. When compared to other serological tests for antibodies, such as immunofluorescence, complement fixation, hemagglutination, and radioimmunoassays, ELISA had similar sensitivity, specificity, and repeatability. The values of serum lgG antibodies less than 0.9 is considered negative, while lgG values more than or equal to 0.9 is considered as positive [24]. As previous studies have reported, the definition of *H* pylori IgG seropositivity is value ≥0.9 in our study [23, 25].

### 2.4. Other Covariates

In our study, we extracted the confounding factors that may have a potential impact on DII and *H. pylori*, including demographic information, lifestyle, self-reported health status, physical examinations, and biochemical results. Demographic information including sex (male/female), age (year), race (Mexican American/other Hispanic/non-Hispanic White/non-Hispanic Black/other races), the ratio of family income to poverty threshold. Drinking status was categorized as never (had <12 drinks in a lifetime), former (had ≥12 drinks in 1 year and did not drink last year, or did not drink last year but drank ≥12 drinks in a lifetime), current light/moderate drinker (≤1 drink per day for women or ≤2 drinks per day for men on average over the past year), or current heavier drinker (>1 drink per day for women or >2 drinks per day for men on average over the past year). Body mass index (BMI, kg/m^2^) were included. Current medical conditions including diabetes (yes/no), hypertension (yes/no), congestive heart disease (yes/no), and stroke (yes/no) that based on self-reported results. Laboratory data included fasting plasma glucose (mg/dl), plasma insulin (pmol/L), alanine transaminase (ALT, IU/L), aspartate transaminase (AST, IU/L), C-reactive protein (mg/dl), triglycerides (mmol/L), and glycated hemoglobin (HbA1c).

### 2.5. Statistical Analysis

All analyses were conducted using R software (4.1.1). Due to the complexity of the NHANES survey's design, we followed the survey's instructions and used a cycle weight, stratification, and clustering for each analysis. Participants were divided into two groups according to *H. pylori* antibody status. Categorical variables were presented as weighted numbers and proportions, while continuous variables were displayed as weighted means with standard errors.

**Figure 2:**
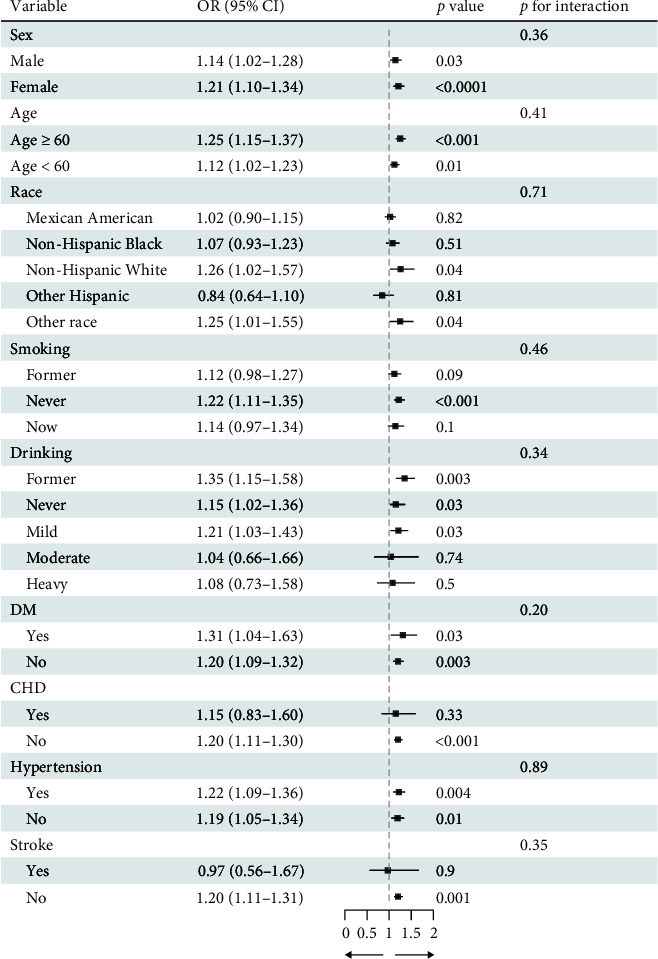
The forest plot of subgroup analysis between DII and *H. pylori* infection. In the subgroup analysis stratified by sex, age, race, smoking, drinking, DM, CHD, hypertension, and stroke, the model is not adjusted for sex, age, race, smoking, drinking, DM, CHD, hypertension, and stroke, respectively.

Using weighted linear regression for continuous variables and the design-adjusted *χ*^2^ test for categorical variables, baseline characteristics were compared across groups. In addition, multiple logistic weighted regression analysis was used to control for potential variables and to generate odds ratios (ORs) and 95% confidence intervals (CIs) in order to examine the association between DII and HP infection status. DII score were presented as continuous variable and categorical variable (divided by the tertile of DII score) in the logistic weighted regression model. Model 1 had no covariate-adjusted; model 2 adjusted age, gender, and race; and model 3 adjusted for gender, age, race, ALT, AST, body mass index, family income to poverty ratio, fasting plasma glucose, plasma insulin, HbA1c, C-reactive protein, triglycerides, hypertension, stroke, diabetes, congestive heart disease, alcohol use, and smoking status. In addition, restricted cubic splines (RCS) with adjustments for potential confounders were used to explore a potential nonlinear connection between DII and *H. pylori* infection status. The *p*-value for the nonlinearity of the smooth curve fitting was calculated. In addition, we performed subgroup analysis and computed the interaction impact. The results of subgroup analysis were shown in forest plot of Figure 2. The forest plot can help visualization and comparison of effect estimates. Statistical significance was determined at a *p*-value of <0.05.

## 3. Results

### 3.1. Baseline Characteristics of Study Population

A total of 4,000 eligible individuals were enrolled in our study. The demographic data and laboratory indexes of the finally enrolled 4,000 participants were presented in Table 1 divided by *H. pylori* infection status. The average age of enrolled participants was 45.92 years, and 46.77% percent were male. In our study, the overall average DII was 0.47 (0.07).The overall prevalence of *H. pylori* seropositive in the study population was 45.9%. Participants with *H. pylori* seropositive were significantly older than *H. pylori* seronegative (49.54 (0.57) vs. 44.17 (0.51), respectively; *P* < 0.0001). No significant sex differences were observed between *H. pylori* positive and negative group. Moreover, participants with *H. pylori* seropositive had significantly higher incidence of diabetes and hypertension.(Table 1). Significant differences between participants with or without *H. pylori* infection were also shown in family income to poverty ratio, smoke, alcohol use, plasma glucose, and HbA1c (Table 1).

### 3.2. Association between DII and H. pylori IgG Seropositivity in US Adults

**Figure 3:**
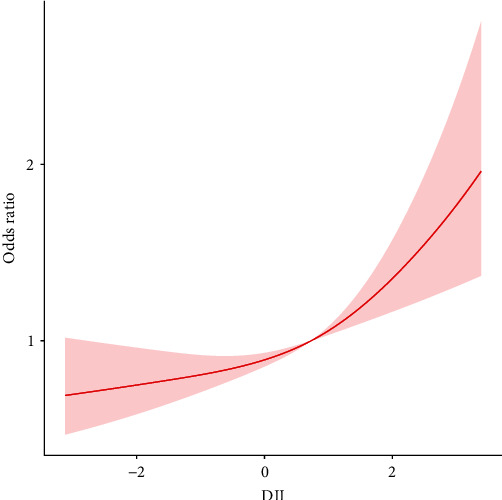
The restricted cubic spline for the associations of DII with *H. pylori* infection based on model 3. Knots were placed at the 5th, 35th, 65th, and 95th percentiles of the DII distribution. The red solid line and red shaded area represent estimated values and their corresponding 95% confidence intervals.

**Figure 4:**
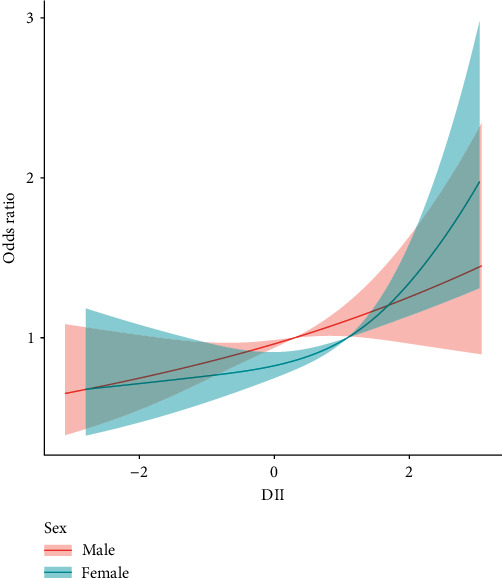
The association between DII and *H. pylori* infection stratified by sex. The shaded areas in orange and green represent the confidence intervals for male and female, respectively. Knots were placed at the 5th, 35th, 65th, and 95th percentiles of the DII distribution.

To evaluate the connection between DII and *H. pylori* IgG seropositivity, three sample-weighted multivariable logistic models were constructed. In Section 2, the potential confounding factors that were adjusted in each model were described. In model 1(OR = 1.18; 95% CI, 1.09–1.28, *p* < 0.001) and model 2 (OR = 1.20; 95% CI, 1.11–1.30, *p* < 0.001), DII score was significantly associated with *H. pylori* IgG seropositivity. In model 3, the ORs were 1.17 (95% CI, 1.09–1.27) indicating that each unit of increased DII score was associated with 17% increased risk of *H. pylori* IgG seropositivity, respectively. To ensure the accuracy of our results, we also performed a sensitivity analysis to compare the relations between DII, as a categorical variable divided by tertile, and *H. pylori* IgG seropositivity. The first tertile group (T1, DII < 0.041), the second one (T2, DII = 0.041–1.32), and the third one (T3, DII > 1.32). The ORs of T3 and T2 are 1.7 (95% CI, 1.27–2.28) and 1.3 (95% CI, 1.03–1.64), respectively, in the fully adjusted model (model 3). The details of logistic results are shown in Table 2. A nonlinear association between *H. pylori* IgG seropositivity and DII was examined by calculating the *P*-value for the trend of the DII score in each model using R software (*p* for trend <0.001), as shown in Table 2. Moreover, after controlling for all confounding variables, the smooth curve fitting revealed a positive and almost linear relationship between DII score and the incidence of *H. pylori* seropositive (Figure 3). In the sex stratification, the near-linear relations between DII and *H. pylori* seropositive were also observed (Figure 4).

### 3.3. Subgroup Analysis

Subgroup analysis was conducted to assess the robustness of our findings within each demographic category. The subgroup-specific results are presented in Figure 2, and the findings were found to be consistent across all subgroups. Notably, we observed a number of particularly strong associations between DII level and *H. pylori* seropositivity in female participants, individuals of non-Hispanic White ethnicity, never smokers, and former alcohol users. Furthermore, we found that individuals with comorbid diabetes mellitus and hypertension were more likely to exhibit a strong association between DII level and *H. pylori* seropositivity. Importantly, no significant interactions between DII level and *H. pylori* seropositivity were identified in the subgroup analyses, as indicated by all interaction *p*-values exceeding 0.05.

## 4. Disscussion

In this present cross-sectional study, we enrolled 4,000 individuals who met our inclusion criteria. We found that higher DII levels were independently associated with an increased risk of *H. pylori* IgG seropositivity, even after adjusting for all potential confounding factors. A near-linear positive association between DII and *H. pylori* IgG seropositivity was observed after smoothing the curve based on RCS. In addition, subgroup analyses and interaction tests revealed that the association between DII and *H. pylori* IgG seropositivity was stable across diverse population situations.

As far as we know, our study is the first to evaluate the relationship between DII and *H. pylori* among US adults. Our findings suggest that diet with higher DII or proinflammatory diet may increase the risk of *H. pylori* infection. There is growing evidence that dietary patterns play a significant role in the modulation of *H. pylori* infection, as it can alter the gastric environment, and many studies have been conducted concerning dietary factors and *H. pylori* infection. A study based on Chinese population found that a diet with higher carbohydrates and sweets could increase the susceptibility to *H. pylori* infection [5]. In that study, researchers also found that high intake of red and processed meat, which are rich in saturated fats, has also been associated with increased risk of *H. pylori* infection [5]. In addition, Animal studies have shown that diets high in salt can promote *H. pylori* colonization by disrupting the integrity and viscosity of the gastric mucosa. This alteration creates a proinflammatory state, which leads to increased epithelial damage, hypochlorhydria, and ultimately gastric cancer [26, 27]. A recent in vitro study has demonstrated that the concentration of salt in the environment has a notable effect on the composition of the *H. pylori* exoproteome. Specifically, the study found that higher salt concentrations led to an increase in the levels of a secreted VacA toxin, which has been linked to an elevated risk of gastric cancer. These findings underscore the potential association between a high-salt diet and the progression of *H. pylori* infection, and highlight the importance of further investigations into the role of diet in the development and prevention of this bacterial infection [28]. Therefore, these studies emphasize the vital role of dietary patterns in the development and progression of *H. pylori* infection.

In this study, we found a significant association between the DII and *H. pylori* infection among various subgroups. In the subgroup analysis, females and older population were more likely to be infected by *H. pylori*. Drawing from the Korean National Health and Nutrition Examination Survey that included 12,095 study participants, an analysis of the data revealed that after controlling for potential confounding variables such as age, sex, BMI, education, aspirin use, stress, and metabolic disease, females were found to be at a higher risk of developing peptic ulcer disease compared to males. In addition, a study based on South Korean population also found that females were more likely to have *H. pylori* infection. The mechanisms underlying the association between proinflammatory diet and *H. pylori* infection remains unclear. One possible explanation is that proinflammatory diet may alter the gut microbiota and decrease the abundance of beneficial bacteria, which may create an environment that is more conducive to the growth of *H. pylori* [1]. In addition, proinflammatory diet may increase oxidative stress and inflammation, which may compromise the immune response to *H. pylori* and facilitate its colonization in the stomach [29]. In addition to the potential mechanism discussed above, other factors such as age, sex, socioeconomic status, and geographical location may also play a role in the association between proinflammatory diet and *H. pylori* infection [2, 30].

Our study has several strengths and limitations. To our knowledge, our study is the first study to assess the association between DII and *H. pylori* infection in the general population. Moreover, we had a large sample size with representative sample selection, which can help adjust for more confounding factors to produce more reliable results. Moreover, by RCS and smooth curve fitting, a near-linear relationship were observed and calculated, which could help us understand the complicated relationship between DII and *H. pylori* infection in general population. But as an observational and cross-sectional study design, our study also has several limitations. First, as a cross-sectional analysis, a definite causal relationship is difficult to obtain, therefore a prospective study is needed. Second, although we have adjusted for several confounding factors, we still cannot completely rule out other confounding factors. Third, we used data from NHANES 1999–2000, about 20 years ago, Although these data may not reflect the current dynamics of dietary patterns, we believe they still hold value for research purposes. By analyzing past data, we can gain preliminary insights into the relationship between *Helicobacter pylori* and dietary factors, laying a foundation for more in-depth investigations in the future. And many studies have shown a gradual increase in the DII index over the past few decades [6, 31, 32]. In our study we found that higher DII was associated with higher risk of *H. pylori* infection. Increased DII in current situation may also associated with higher rate of *H. pylori* infection at present. Our study can lay a foundation for further prospective study in the context of current dietary patterns. Further researches are necessary to elucidate the exact mechanisms of DII and *H. pylori* infection in this population.

## 5. Conclusion

In conclusion, our study provides preliminary evidence for the association between high DII score diet and *H. pylori* infection. Further studies with larger sample size and more comprehensive assessment of dietary intake and other potential confounding factors are needed to confirm and extend our findings. Our results may have important implications for the prevention and management of *H. pylori*-related diseases, and highlight the importance of a healthy diet in maintaining gastrointestinal health.

## Figures and Tables

**Table 1:** Baseline characteristics of study population between *H* pylori seropositive group and negative group, weighted.

Variable	Total	HP seronegative	HP seropositive	*P*-value
Age (year)	45.92 (0.41)	44.17 (0.51)	49.54 (0.57)	<0.0001
Sex (%) (SE)				0.94
Female	2,129 (53.23)	1,184 (51.88)	945 (51.99)	
Male	1,871 (46.77)	979 (48.12)	892 (48.01)	
Race (%) (SE)				<0.0001
Mexican American	1,097 (27.42)	350 (4.01)	747 (14.11)	
Non-Hispanic Black	721 (18.02)	299 (6.86)	422 (17.09)	
Non-Hispanic White	1,806 (45.15)	1,352 (80.86)	454 (48.51)	
Other Hispanic	258 (6.45)	100 (5.12)	158 (14.35)	
Other race	118 (2.95)	62 (3.15)	56 (5.94)	
Smoke, % (SE)				0.01
Former	1,071 (26.83)	580 (25.78)	491 (25.27)	
Never	2,118 (53.06)	1,176 (52.85)	942 (46.08)	
Now	803 (20.12)	403 (21.36)	400 (28.65)	
Alcohol (%) (SE)				<0.001
Former	793 (20.74)	369 (14.47)	424 (22.06)	
Heavy	723 (18.91)	385 (20.68)	338 (21.08)	
Mild	1,208 (31.59)	726 (35.87)	482 (28.58)	
Moderate	515 (13.47)	318 (17.33)	197 (13.96)	
Never	585 (15.3)	297 (11.64)	288 (14.32)	
Hypertension (%)	1,791 (44.77)	884 (34.85)	907 (43.42)	0.003
Diabetes (%)	518 (13.77)	209 (7.12)	309 (12.26)	0.002
Coronary heart disease (%)	158 (3.97)	75 (2.79)	83 (4.31)	0.09
Stroke (%)	129 (3.23)	58 (2.03)	71 (3.00)	0.09
Family income to poverty ratio	2.91 (0.12)	3.16 (0.13)	2.38 (0.10)	<0.0001
BMI (kg/m^2^)	28.04 (0.22)	27.99 (0.28)	28.14 (0.16)	0.59
Fasting plasma glucose (mg/dl)	100.75 (0.99)	98.40 (1.07)	105.86 (1.88)	0.002
Plasma insulin (pmol/L)	77.33 (2.58)	75.86 (3.08)	80.51 (2.90)	0.19
HbA1c	5.42 (0.04)	5.31 (0.04)	5.63 (0.05)	<0.0001
ALT, (IU/L)	26.55 (0.48)	26.50 (0.70)	26.64 (0.62)	0.89
AST, (IU/L)	24.68 (0.38)	24.54 (0.55)	24.98 (0.50)	0.6
Triglycerides (mmol/L)	1.64 (0.04)	1.59 (0.06)	1.75 (0.05)	0.07
C-reactive protein (mg/dl)	0.44 (0.01)	0.42 (0.02)	0.47 (0.02)	0.09
DII	0.47 (0.07)	0.36 (0.09)	0.69 (0.06)	<0.001

BMI, body mass index; ALT, alanine transaminase; AST, aspartate transaminase; HbA1c, glycosylated hemoglobin; DII, dietary inflammatory index.

**Table 2:** Association between DII and HP infection status among US adults in NHANES 1999–2000—sample weighted multivariate logistic analysis.

Variable	OR (95% CI), *P*-value		
	Model 1	Model 2	Model 3
DII as a continuous variable	1.18 (1.09, 1.28), <0.001	1.20 (1.11, 1.30), <0.001	1.17 (1.09, 1.27), <0.001
DII tertile			
T1	Reference	Reference	Reference
T2	1.39 (1.18, 1.63), <0.001	1.34 (1.08, 1.65), 0.01	1.30 (1.03, 1.64), 0.03
T3	1.73 (1.31, 2.29), <0.001	1.81 (1.33, 2.47), 0.003	1.70 (1.27, 2.28), 0.002
*p* for trend	<0.001	<0.001	<0.001

Model 1: no adjustment. Model 2: adjusted for age, gender, and race. Model 3: adjusted for gender, age, race, ALT, AST, body mass index, family income to poverty ratio, fasting plasma glucose, plasma insulin, HbA1c, triglycerides, C-reactive protein, hypertension, stroke, diabetes, CVD, alcohol use, and smoking status.

## Data Availability

Publicly available datasets were analyzed in this study. This data can be found here: https://www.cdc.gov/nchs/nhanes.

## Supplementary Materials

Supplementary Materials In the supplementary material, we described the detailed calculation process of DII and shown the clinical significance of DII. In Table S1, we described the mean intake of 28 dietary components for DII calculation.
