# Supplementary material for: Association of Dietary Inflammation Index and Helicobacter pylori Immunoglobulin G Seropositivity in US Adults: A Population-Based Study
Source: Mediators Inflamm. 2023 Jul 28;2023:8880428. doi: 10.1155/2023/8880428 (PMC10403320; doi:10.1155/2023/8880428)
Supplement: Supplementary Materials — In the supplementary material, we described the detailed calculation process of DII and shown the clinical significance of DII. In Table S1, we described the mean intake of 28 dietary components for DII calculation. [file 8880428.f1.docx]

**Supplementary material for the article:**

**1. The general formulation of DII calculation**

In fact the calculation of DII does not have an exact mathematical formula[1]. However, in the previous studies, the creators and users of DII have described the summarized formulation of DII calculation[1, 2]. The calculation of DII can be expressed by the following abstract formula:

DII = Σ (Z_i x W_i)

In this formula, i represents each food or nutrient, Z_i represents the standardized value for each food or nutrient, and W_i represents the respective inflammatory effect score for each food or nutrient.

The calculation steps are as follows:

1.Compilation of data: Gather data on the inflammatory effect scores for various foods and nutrients from scientific literature. **(Step 1 in flow chart)**

2.Standardization: Standardize the values for each food or nutrient using a specific formula. The standardized value (Z_i) is calculated as (X_i - B_i) / SD_i, where X_i is the raw intake value of the food or nutrient, B_i is the global mean, and SD_i is the standard deviation of global mean. **Detailed process is shown in flow chart (step 5-6).**

3.Weighting and summation: Assign weights (W_i) to each food or nutrient based on the strength of its association with inflammation. These weights are derived from scientific literature. Multiply each standardized value (Z_i) by its respective weight (W_i), and sum up the products for all foods and nutrients to obtain the overall DII score. **(Step 2-4 and 7-8 in flow chart)**

By applying this formulation and following the calculation steps, the DII can be computed for an individual, providing an assessment of the inflammatory potential of their diet.

**The detailed methodology of standardization as shown below**[1]. In general standardized value (Z_i) or Z score is by subtracting the global daily average intake of a specific dietary component from the daily average intake of that component in a particular diet, and then dividing it by the standard deviation of the global daily average intake of that component. To minimize the effect of ‘right skewing’, this value is converted to a percentile score. To achieve a symmetrical distribution with values centred on 0 (null) and bounded between −1 (maximally anti-inflammatory) and +1 (maximally pro-inflammatory), each percentile score is doubled and then ‘1’ is subtracted.

**2. The flow chart of DII calculation**

The flow chart of calculating DII can help us understand the calculation process quickly and easily. The step 1-5 had done by the authors of DII. Therefore, we just need to obtain individual dietary data and continue the step 6-8.


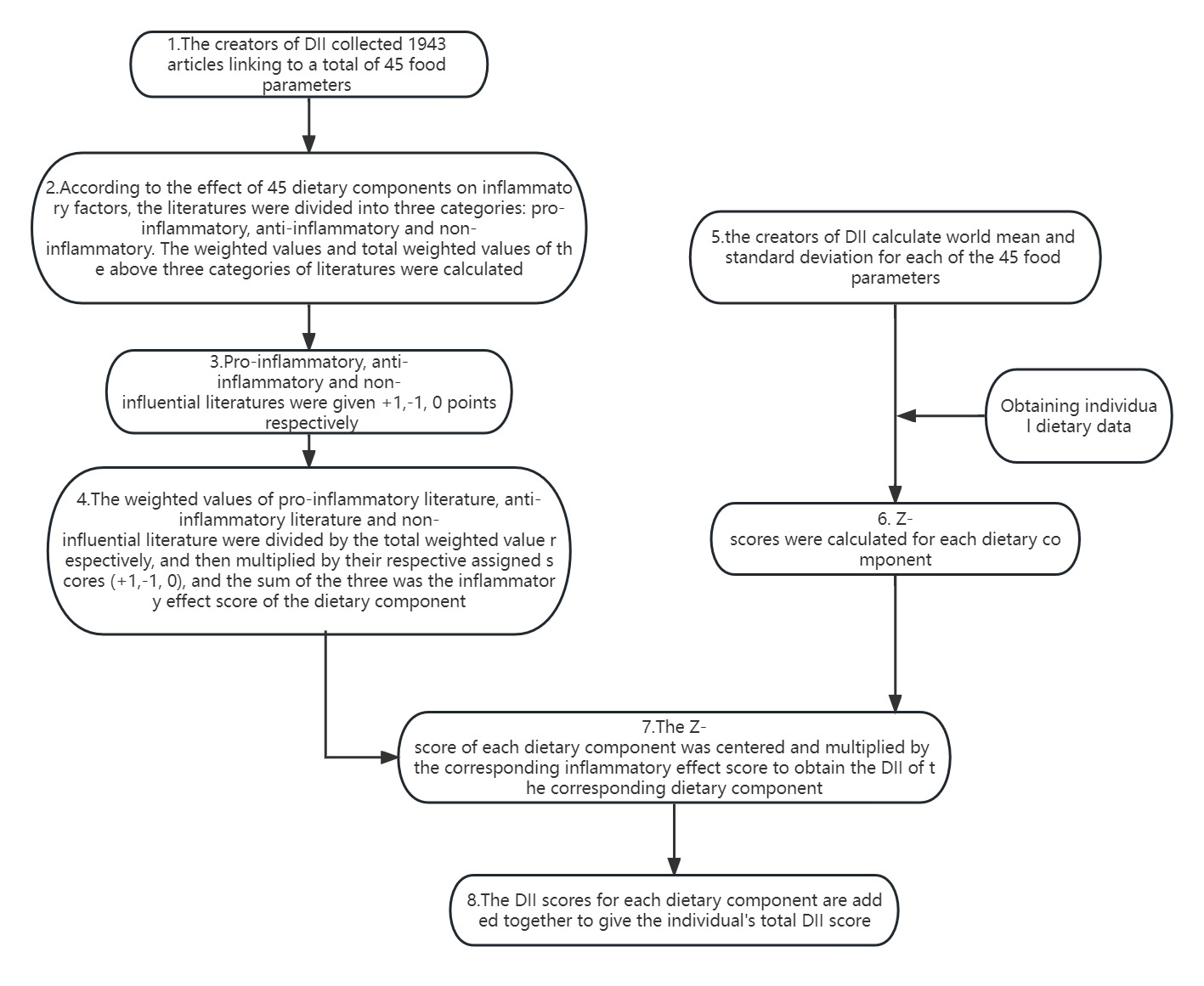


Supplementary table 1 Food parameters included in the dietary inflammatory index

| Food parameter | Mean intake |
| --- | --- |
| Carbohydrate (g) | 219.6 |
| Energy (kcal) | 220.7 |
| Protein(g) | 1933.33 |
| Fat(g) | 78.98 |
| Fibre(g) | 68.16 |
| Cholesterol (mg) | 19.25 |
| SFA(g) | 299.07 |
| MUFA (g) | 21.02 |
| PUFA (g) | 26.71 |
| β-carotene(ug) | 17.78 |
| Vitamins A (RE) | 2100.01 |
| Vitamins B1 (mg) | 589.49 |
| Vitamins B2 (mg) | 1.38 |
| Vitamins B6(mg) | 1.70 |
| Vitamins B12(μg) | 1.58 |
| Vitamins C (mg) | 4.11 |
| Vitamins D (μg) | 62.07 |
| Vitamins E (mg) | 3.5 |
| Folic acid (ug) | 9.32 |
| Niacin(mg) | 19.81 |
| Fe (mg) | 360.36 |
| Mg (mg) | 16.76 |
| Zn (mg) | 270.68 |
| Se(μg) | 11.13 |
| n-3 PUFA (g) | 111.15 |
| n-6 PUFA (g) | 1.75 |
| Alcohol (g) | 13.89 |
| Caffeine (g) | 4.91 |

1. Shivappa N, Steck SE, Hurley TG, Hussey JR, Hebert JR: **Designing and developing a literature-derived, population-based dietary inflammatory index**. *Public Health Nutr* 2014, **17**(8):1689-1696.

2. Zhao L, Sun Y, Liu Y, Yan Z, Peng W: **A J-shaped association between Dietary Inflammatory Index (DII) and depression: A cross-sectional study from NHANES 2007-2018**. *J Affect Disord* 2023, **323**:257-263.
